# Quantifying the Accuracy of Digital Hemispherical Photography for Leaf Area Index Estimates on Broad-Leaved Tree Species

**DOI:** 10.3390/s18041028

**Published:** 2018-03-29

**Authors:** Carlo Gilardelli, Francesca Orlando, Ermes Movedi, Roberto Confalonieri

**Affiliations:** 1 Cassandra Lab, Università degli Studi di Milano, DESP, via Celoria 2, I-20133 Milan, Italy; ermes.movedi@unimi.it; 2 Department of Agricultural and Environmental Sciences—Production, Landscape, Agroenergy, Università degli Studi di Milano, DESP, via Celoria 2, I-20133 Milan, Italy; francesca.orlando@unimi.it

**Keywords:** digital hemispherical photography, leaf area index, precision, trueness, woody canopies

## Abstract

Digital hemispherical photography (DHP) has been widely used to estimate leaf area index (LAI) in forestry. Despite the advancement in the processing of hemispherical images with dedicated tools, several steps are still manual and thus easily affected by user’s experience and sensibility. The purpose of this study was to quantify the impact of user’s subjectivity on DHP LAI estimates for broad-leaved woody canopies using the software Can-Eye. Following the ISO 5725 protocol, we quantified the repeatability and reproducibility of the method, thus defining its precision for a wide range of broad-leaved canopies markedly differing for their structure. To get a complete evaluation of the method accuracy, we also quantified its trueness using artificial canopy images with known canopy cover. Moreover, the effect of the segmentation method was analysed. The best results for precision (restrained limits of repeatability and reproducibility) were obtained for high LAI values (>5) with limits corresponding to a variation of 22% in the estimated LAI values. Poorer results were obtained for medium and low LAI values, with a variation of the estimated LAI values that exceeded the 40%. Regardless of the LAI range explored, satisfactory results were achieved for trees in row-structured plantations (limits almost equal to the 30% of the estimated LAI). Satisfactory results were achieved for trueness, regardless of the canopy structure. The paired *t*-test revealed that the effect of the segmentation method on LAI estimates was significant. Despite a non-negligible user effect, the accuracy metrics for DHP are consistent with those determined for other indirect methods for LAI estimates, confirming the overall reliability of DHP in broad-leaved woody canopies.

## 1. Introduction

Leaf area index (LAI; total one-sided area of leaf tissue per unit ground surface) is widely recognized as a key variable for a broad range of agro-environmental studies, given its tight relationships with primary production [1], transpiration [2], energy exchange [3], CO_2_ sequestration [4] and with a variety of other eco-physiological processes [5]. However, the direct measurement of LAI is often unfeasible, especially in operational context or when woody canopies are involved [6]. In these cases, LAI is usually estimated using indirect methods, based on the measurement of the amount of radiation (i) reflected/absorbed by the canopy (remote sensing techniques) or (ii) transmitted through it (optical proximal instruments). The digital hemispherical photography (DHP)—like other methods based on image processing (e.g., PocketLAI, [4])—can be classified in the second category, although it derives gap fraction from image segmentation instead of using the above-to-below canopy luminance ratio. DHP is one of the less expensive techniques and gives to users the possibility to reprocess archives (e.g., correction of incorrect exposure, removal of unwanted objects) because of its permanent image recording. For these reasons and for the partial suitability of other indirect methods, it is widely used for LAI estimates in orchards and forest stands [7,8,9,10]. However, it is more time-consuming than other methods such as those based, for example, on LiDAR [11,12] because of the asynchronous and not automated image processing, which anyhow provides information to fully characterize the canopy in terms of openness and foliage density [13,14,15].

After acquisition, hemispherical images are processed using dedicated software packages (e.g., GapLightAnalyzer, Forest Renewal BC, Can-Eye), which classify pixels into two categories based on thresholds defined by the user through trial and error adjustments. Despite the widespread use of this method in forestry, some constraints potentially limit its reliability. Examples are the influence of camera exposure (e.g., [16,17]), the dependency on the view zenith angle [18] and the selection of the optimal segmentation thresholds. The latter is often considered as one of the most crucial issues [6], given the high level of subjectivity due to the user’s sensibility and experience [19]. Moreover, threshold identification can be influenced by the pixel category (i.e., sky or green) from which the user decides to start [20]. For these reasons, the impact of these user-dependent factors on the accuracy of DHP LAI estimates constitutes an important issue that deserves to be thoroughly investigated. According to ISO (1994), accuracy is composed by trueness and precision, the first being the agreement between real values and the means of replicated measurements, the latter being the agreement within a series of replicated measurements, performed by the same user (repeatability) or by independent users under different conditions (reproducibility) [21].

This study aimed at quantifying both the components of the accuracy of DHP, providing practical information for LAI estimates in broad-leaved tree species. Regarding precision, the limits of repeatability and reproducibility were computed. Concerning the trueness, the unfeasibility of performing direct LAI measurements in tree canopies suggested to estimate it based on artificial canopy images, as already performed by [22,23].

## 2. Materials and Methods

Data used for precision determination—described in detail by [10]—were acquired using a smartphone Samsung GT-i9105 Galaxy S II Plus equipped with a fisheye lens AKASHI ALTLENS4IN1SG2. In the current study, only the images collected by [24] on broad-leaved canopies were used. Images were acquired using two different protocols: for row-structured plantations, images were acquired moving parallel to the row, whereas in case of sparse or continuous canopies, they were acquired moving around the trunk at a distance of approximately half crown radius. All images were acquired by positioning the lens upwards, avoiding the inclusion of direct sunlight in the images. The software used for image processing was Can-Eye (v. 6.314; www.avignon.inra.fr/can_eye; [25]), which allowed obtaining the best results in an extensive comparative study [9]. Precision metrics were determined by following the adaptation of the ISO 5725 protocol [26] to in vivo field methods proposed by [21]. To estimate the repeatability and reproducibility of DHP, three classes of broad-leaved tree species were considered (Table 1).

This choice allowed complying with the ISO 5725 protocol, which indicates to perform the analysis on different batches of materials. Within each class, four plants were selected on the basis of the mean LAI values estimated by [10], by dividing LAI data into quartiles (25, 50, 75, 100%) and choosing one set of images within each quartile, in order to explore a wide ranges of LAI values (i.e., levels in the ISO 5725 protocol).

The images for each of the entities reported in Table 1 were processed by four independent users (laboratories A, B, C and D hereafter, according to the ISO 5725 terminology) already involved in studies targeting LAI estimates from DHP using the Can-Eye software and so autonomous and independent. Each user repeated the procedure for LAI estimates four times, letting 10 days pass between times (four measurement replicates for each entity and for each user), leaving them free choice for the Can-Eye segmentation method and threshold identification. According to ISO 5725, possible outliers were identified by applying the Cochran’s ([27], Equation (1)) and Grubbs’ ([28], Equation (2)) tests, targeting outlying values detection, respectively, in variances and means from different laboratories.

(1) $$C=\frac{S_{\max}^{2}}{\sum_{1=1}^{n}S_{i}^{2}}$$

where C is Cochran’s test value, $S_{\max}^{2}$ is maximum variance among those calculated for the different laboratories and $S_{i}^{2}$ is the variance of the ith laboratory.

(2) $$G=\frac{\left|\mathrm{suspect}\;\mathrm{value}-\;\overset{\text{\_}}{x}\right|}{S}$$

where G is the Grubbs’ test value, $\overset{\text{\_}}{x}$ is mean of all the estimates, suspect value is the highest (or lowest) laboratory mean and S is the standard deviation of the values measured by the different laboratories.

According to the relationships between the test metrics and related tabulated values, laboratories were considered as non-outlying, stragglers (not enough different from the others and thus maintained in the following steps of the analysis; the test result is greater than its 5% critical value) or outliers (excluded; the test result is greater than its 1% critical value) [26]. Repeatability and reproducibility (as well as their relative values and limits, [26]; Equations (3) and (4)) were quantified by targeting, respectively, the four sequential replicates from the same user within each laboratory and all LAI values estimated by the laboratories under reproducibility conditions. Thus, obtaining the maximum range of variation between two measures performed by the same (repeatability limit) operator or by different (reproducibility limit) operators [21].

(3) $$r=S_{r}\cdot \sqrt{2}\cdot t$$

(4) $$R=S_{R}\cdot \sqrt{2}\cdot t$$

where r = repeatability limit; R = reproducibility limit; S_r_ = standard deviation of repeatability; S_R_ = standard deviation of reproducibility; t = two-tailed critical value of the Student t distribution at the 95% confidence level for infinite freedom degrees [26].

Because of the unfeasibility of measuring LAI in woody canopy using direct methods, DHP trueness was quantified using artificial images with known canopy cover, adapting the procedure proposed by [3]. These images were obtained through the random generation of 3 mm radius black circles, using an R routine (R Core Development Team, Vienna). The choice for the 3 mm radius circles was dictated by the necessity to obtain representative images of canopy cover while containing the computational costs for their generation. Fourteen sets of images were generated with canopy covers ranging from 30% to 85% to get a representative range of broad-leaved canopy densities. Images were than printed, placed on a levelled surface and acquired using the same set smartphone—fish-eye lens described above. The trueness was then quantified by using indices of agreement between the means of four measurement replicates and the reference values (from the images with known canopy cover). In particular, the trueness was quantified using the coefficient of determination (R^2^) and the relative root mean square error (RRMSE, obtained as the ratio between RMSE and the mean of reference values).

Then, the uncertainty in LAI estimates due to the selection of different segmentation methods was quantified by considering the whole 126-item dataset of tree broad canopy images from [10]. To avoid mixing different sources of uncertainty, a single user processed all the images in this phase, using alternatively the two segmentation methods (based on sky or green pixel detection). The methods differ from the pixel’s category on which the threshold determination is based. For instance, using the green method, the user adjusts the threshold for image segmentation trying to discriminate the pixels belonging to green surfaces from the pixels belonging to background.

A paired *t*-test was used to assess the significance of the differences due to the segmentation method.

## 3. Results and Discussion

Table 2 shows the results of the statistical analyses and the means of the estimates (less than those identified as outliers) performed on the three categories of tree canopies to derive DHP precision metrics. The Grubbs’ test led to identify one value as outlier (laboratory C for *Magnolia grandiflora* L.) and two values as stragglers (laboratories B and C for *Olea europaea* L.), whereas the Cochran’s test led to identify laboratory B as outlier for *Populus* spp. Outliers were not used for repeatability and reproducibility determination.

**Figure 1:**
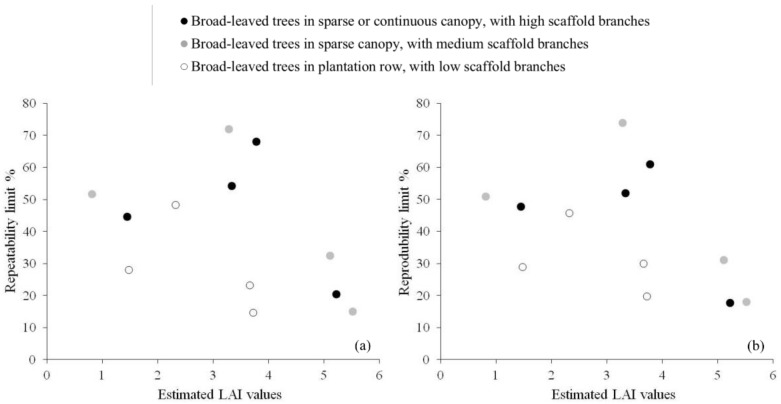
Relationship between repeatability (**a**) and reproducibility (**b**) limits of LAI estimates and mean LAI values for different canopy classes. Limits of repeatability and reproducibility were reported as a percent of the related estimated value. Black circles refer to broad-leaved trees in sparse or continuous canopy, with high scaffold branches; grey circles refer to broad-leaved trees in sparse canopy, with medium scaffold branches; white circles refer to broad-leaved trees in plantation row, with low scaffold branches.

In general, the best values for repeatability and reproducibility limits with respect to the estimated LAI were achieved for LAI higher than 5 (Figure 1), corresponding to the 4th quartile of each canopy class (Table 1).

For this range of LAI values, indeed, mean values for repeatability and reproducibility limits were equal to 1.18, roughly corresponding to 22% of the estimated LAI. Results for medium (between 2.5 and 4) and low LAI values (lower than 1.5) were instead less satisfactory. For the intermediate LAI values, the average repeatability and reproducibility limits were equal to 1.54 and 1.56, corresponding to about 47% of the estimated LAI. Precision metrics were slightly better for low LAI values, with mean repeatability and reproducibility limits equal to 0.50 and 0.51 (almost 42% of the estimated values).

These results suggest that, when hemispherical images were composed by a prevalent proportion of vegetation (high LAI values), the impact of the uncertainty in threshold definition during segmentation was negligible. In these cases, indeed, the low relative presence of mixed pixels (or of image regions characterized by sky and vegetation pixels mixed up with a fine level of granularity) because of a large proportion of vegetation, simplified the determination of threshold values both between and within laboratories. On the contrary, when hemispherical images were either evenly composed by sky and vegetation pixels (intermediate LAI values) or characterized by a high proportion of sky (low LAI values), the determination of the threshold value—and thus the image segmentation—was more affected by the user sensibility and experience, because of a larger number of pixels potentially subject to uncertain classification. An exception to the positive relationship between mean LAI values and precision metrics was observed for broad-leaved trees in plantation row. For this class, an intermediate value of the precision metrics was achieved, regardless of the mean LAI value (Table 2). Mean repeatability and reproducibility limits were indeed equal to 0.73 and 0.83, respectively (28.5% and 31.1% of the estimated LAI) and the corresponding relative standard deviations did not exceed 11.4%. Probably, the continuity of the canopy guaranteed by the planting layout and the position of branches (all the trees belonging to this canopy class were cultivated as short rotation forestry), as well as the protocol followed for images acquisition [10], allowed obtaining close estimates both within and between laboratories, independently from the LAI values.

The values of precision metrics achieved in this study for DHP are slightly larger than those estimated—on homogeneous rice crops—by [4] for the smart app PocketLAI and for the AccuPAR ceptometer (Decagon, Pullman, WA, USA). The mean values of repeatability and reproducibility limits for DHP determined in this study (including all the canopy classes) were 1.19 and 1.25, respectively, whereas they were 0.80 and 0.82 for PocketLAI and 0.73 and 0.82 for AccuPAR. Also, the variability among the precision metrics calculated for different samples for DHP was practically the same of that estimated for AccuPAR, whereas PocketLAI presented a larger variability. DHP achieved instead values for the precision metrics that were slightly better than those estimated for LAI-2000 (Decagon, Pullman, WA, USA), regardless of the instrument 4- or 5-ring configuration [4].

**Figure 2:**
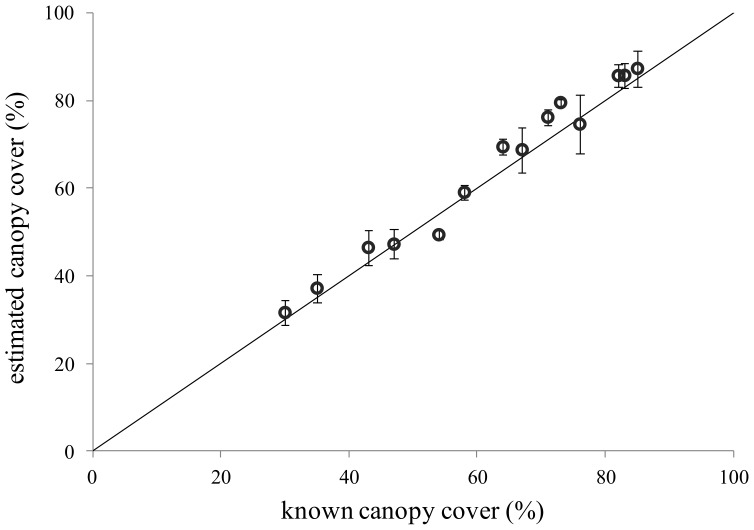
Comparison between LAI values estimated with digital hemispherical photography (DHP) (mean of four replicates) and the reference values (artificial images with known canopy cover). Error bars indicate the standard deviation of the replicates. Black continuous line indicates the perfect agreement between estimates and reference values.

Satisfactory results were achieved in terms of trueness (Figure 2), with estimates always very close to the reference values (i.e., the virtual images generated with a known canopy cover; [3]).

The good agreement between estimates and reference values is confirmed by the values of the performance metrics, with R2 and RRMSE achieving the values of 0.98 and 6%, respectively. Despite this it is worth underlining that our trueness metrics were obtained from artificially generated images, they are better than those achieved by other authors with methods for LAI estimates based on image segmentation (e.g., [4]). The same consideration is valid for the trueness metrics reported for DHP by [30,31] for forest canopies and by [24] for vineyards.

**Figure 3:**
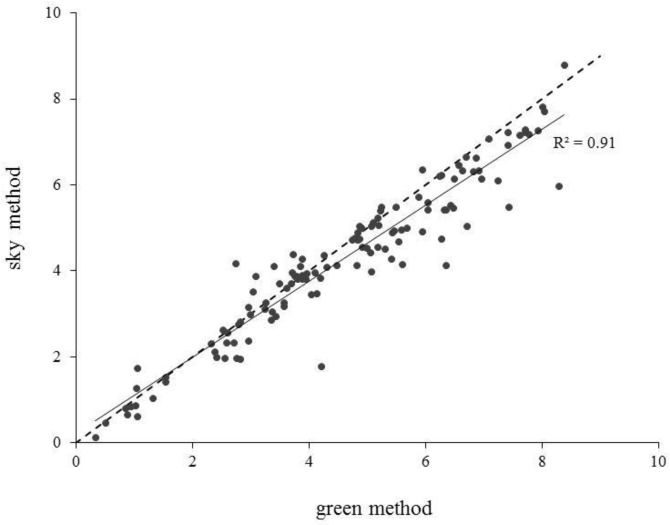
Comparison of LAI estimates obtained by the same user for 126 DHP images using the ‘sky’ and ‘green’ segmentation methods available in Can-Eye.

Figure 3 shows the comparison of LAI estimates obtained—for the whole dataset of DHP images collected by [10]—by the same user with the two segmentation methods (sky or green) available in the Can-Eye software.

In more than 30% of the cases, the two segmentation methods led to differences in LAI estimates that exceeded 0.5, thus indicating that—beside the selection of thresholds—the choice of the segmentation method explain a large part of the variability in LAI estimates due to user subjectivity. This is confirmed by the paired *t*-test, which revealed that the differences between the two series of LAI estimates were significant (*p* < 0.001). In general, a tendency to estimate lower LAI values with the sky segmentation method was observed, likely due to the difficulty in threshold determination for the segmentation of pixels in the external profile of the canopies. Indeed, the corresponding portions of images were often characterized by canopy pixels that appeared very light because of light beams penetrating the vegetation. In these cases, the use of the sky method led to defining threshold values that segmented vegetation pixels as sky, thus determining lower LAI values compared to what achieved using the green segmentation method.

## 4. Conclusions

We quantified the sources of uncertainty in DHP LAI estimates due to user’s subjectivity by calculating the repeatability and reproducibility of the segmentation procedure as offered by Can-Eye, the most popular software for the processing of DHP images. Moreover, we quantified the trueness for DHP using artificial images with known canopy cover. Although we observed a significant effect of the segmentation method selected by the user, our study proved—once more—the reliability of LAI estimates obtained with DHP. Indeed, this technique obtained values for trueness metrics close to their optima and values of repeatability and reproducibility consistent with those of the smart app PocketLAI and of the AccuPAR ceptometer and slightly better than those calculated for LAI-2000. The consistency in LAI estimates both within and between operators, encourage the adoption of such technique to estimate LAI when its direct measurement is unfeasible or to validate other indirect methods (e.g., remote sensing applications). However, some criticalities emerged during this study, particularly regarding the high uncertainty in LAI estimates when the images were characterized by many mixed pixels. These results underline the need to further efforts to better standardize the procedure for LAI estimates from DHP.

## Figures and Tables

**Table 1:** Tree canopies used for determining digital hemispherical photography precision.

ID Class (As in Orlando et al., 2015)	Canopy Class	Species for Each Quartile within the Class
4	Broad-leaved trees in sparse or continuous canopy, with high scaffold branches	*Populus* spp.
		*Liriodendron tulipifera* L.
		*Fagus sylvatica* L.
		*Magnolia grandiflora* L.
3	Broad-leaved trees in plantation row, with low scaffold branches	*Populus* spp.
		*Robinia pseudoacacia* L.
		*Populus* spp.
		*Populus* spp.
1	Broad-leaved trees in sparse canopy, with medium scaffold branches	*Olea europaea* L.
		*Sorbus domestica* L.
		*Acer platanoides* L.
		*Fagus sylvatica* L.

**Table 2:** Precision (repeatability and reproducibility) of digital hemispherical photography (Can-Eye software) in estimating leaf area index (LAI) in tree species. r: repeatability limit; RSDr: relative standard deviation of repeatability; R: reproducibility limit; RSDR: relative standard deviation of reproducibility. In case r was larger than R, R was set equal to r [29].

Canopy Class	Species	Estimated Values	Repeatability		Reproducibility	
			r	RSDr	R	RSDR
Broad-leaved trees in sparse or continuous canopy, with high scaffold branches	*Populus* spp.	1.45	0.65	15.89	0.69	17.04
	*Liriodendron tulipifera* L.	3.78	2.57	24.26	2.57	24.26
	*Fagus sylvatica* L.	3.33	1.81	19.35	1.81	19.35
	*Magnolia grandiflora* L.	5.22 ^a^	1.06	7.09	1.06	7.09
Broad-leaved trees in plantation row, with low scaffold branches	*Populus* spp.	1.48 ^b^	0.41	9.99	0.43	10.32
	*Robinia pseudoacacia* L.	2.32	1.12	17.24	1.12	17.24
	*Populus* spp.	3.66	0.85	8.27	1.09	10.69
	*Populus* spp.	3.72	0.55	5.25	0.73	7.03
Broad-leaved trees in sparse canopy, with medium scaffold branches	*Olea europaea* L.	0.82 ^c^	0.42	18.44	0.42	18.44
	*Sorbus domestica* L.	3.29	2.36	25.68	2.43	26.35
	*Acer platanoides* L.	5.11	1.66	11.58	1.66	11.58
	*Fagus sylvatica* L.	5.52	0.83	5.37	0.99	6.41

^a^ Laboratory C is an outlier according to the Grubbs’ test; ^b^ Laboratory B is an outlier according to the Cochran’s test; ^c^ Laboratory B and C are stragglers according to the Grubbs’ test.
